# A novel biosimulation task trainer for the deliberate practice of resuscitative hysterotomy

**DOI:** 10.1186/s41077-018-0078-1

**Published:** 2018-10-04

**Authors:** Lawrence Lau, Dimitrios Papanagnou, Elaine Smith, Crystal Waters, Elizabeth Teixeira, Xiao Chi Zhang

**Affiliations:** 1 Department of Emergency Medicine, Thomas Jefferson University, 1020 Sansom St., Thompson Bldg, Suite 1651, Philadelphia, PA USA; 2 Sidney Kimmel Medical School, Thomas Jefferson University, 1020 Sansom St., Thompson Bldg, Suite 239, Philadelphia, PA USA; 3 University Clinical Skills and Simulation Center, Thomas Jefferson University, 1001 Locust Street, Suite 409F, Philadelphia, PA USA

**Keywords:** Resuscitation, Resuscitative hysterotomy, C-section, Emergency medicine, Rapid cycle deliberate practice, Task trainer, Education

## Abstract

Resuscitative hysterotomy is a daunting and rarely performed procedure in the emergency department (ED). Given the paucity of clinical exposure to this intervention, resuscitative hysterotomy is an ideal opportunity for simulation-mediated deliberate practice. The authors propose a novel training program using a homegrown, realistic, simulation device as a means to practice resuscitative hysterotomy. Two high-fidelity, tissue-based task-trainer models were constructed and tested on a convenience sample of 14 emergency medicine (EM) residents. The simulated human placenta, bladder, amniotic sac, and uterus were constructed through the use of porcine skin, porcine stomach, transparent plastic bag, Foley tubing, and squid mantle, all secured with nylon sutures. A Gaumard S500 Articulating Newborn was inserted in the simulated uterus, and the entire model was placed into a Gaumard S500 Childbirth Simulator. Each model required less than 1 h for assembly. Emergent hysterotomy was first demonstrated by an EM faculty facilitator, followed by hands-on deliberate practice. Formal feedback on the learners’ self-reported confidence and satisfaction levels was solicited at the end of the workshop through a survey previously cited for use with a low-fidelity resuscitative hysterotomy. Quantitative evaluation of the simulated training session was extracted through a 5-item questionnaire using a 5-point Likert-type scale (i.e., from 1, strongly disagree, to 5, strongly agree). Item scores were added for a cumulative total score, with a possible maximum score of 25 and minimum score of 5. Responses were overwhelmingly positive [24.13 (± 1.36)]. Qualitative feedback was extracted from the survey through open-ended questions; these responses highlighted learners’ appreciation for hands-on practice and the development of a novel, tissue-based simulation task trainer. All participants recommended the training session be available to future learners. Resuscitative hysterotomy is a high-stakes, low-frequency procedure that demands provider practice and confidence. Our hybrid, tissue-based hysterotomy model represents a feasible opportunity for training. The model is cost conscious, easily reproducible, and portable and allows for ample deliberate practice.

**Electronic supplementary material:**

The online version of this article (10.1186/s41077-018-0078-1) contains supplementary material, which is available to authorized users.

## Introduction

The perimortem cesarean section, rebranded in recent years as the “resuscitative hysterotomy,” is perhaps the most daunting and infrequently performed procedure by emergency physicians, necessitating the frequent review of indications, techniques, and pitfalls to ensure the best possible outcome for mother and baby. This procedure is indicated within 5 min of maternal cardiac arrest with a uterine fundus above the umbilicus (indicating a gestation of > 20 weeks) to maximize the probability of favorable maternal neurologic outcome and the secondary goal of fetal survival [1–3]. Despite the importance of resuscitative hysterotomy, the exact circumstances in which this procedure is to be performed are seldom encountered. Given the paucity of clinical exposure to this intervention, resuscitative hysterotomy is an ideal candidate for simulation-mediated deliberate practice.

To date, there are only a few commercially manufactured resuscitative hysterotomy task trainers available for training, further complicated by costly components, maintenance costs, and task trainer availability [4–6]. To address these issues, the authors propose a novel training program using a homegrown, high-realism, task trainer that can be easily inserted into an inexpensive childbirth simulator to teach emergency medicine (EM) residents resuscitative hysterotomy. To further augment the realism of this model, the authors chose to design a lifelike task trainer that provided learners with the realistic feel of organic tissue. Through this program innovation, the authors sought to (1) enhance procedural training of resuscitative hysterotomies; (2) integrate a realistic simulation model for hands-on, rapid cycle deliberate practice (RCDP) with EM residents; and (3) compare the efficacy of our task trainer with similar hysterotomy models for resident learners. RCDP represents a conceptual model for skill acquisition that provides for timely and focused education [7]. Simulation training of high-stakes, low-frequency procedures, combined with RCDP, offers a ripe opportunity for the development of “muscle memory” and enhanced confidence during highly stressful conditions [8]. Through the use of a survey previously cited in the simulation literature [6], the authors aimed to specifically measure the following: preference of instructional delivery with regard to hysterotomy training, appreciation for human anatomic representation, applicability to clinical practice, comfort with performing resuscitative hysterotomy, and familiarity with the procedure. These were each captured through items on the survey.

## Method

For this training program innovation, two identical high-fidelity, tissue-based task-trainer models were constructed and tested on a convenience sample of 14 EM residents at the Thomas Jefferson University, a 3-year EM residency program in Philadelphia, PA, USA. Participant breakdown included five post-graduate-year (PGY)-1 residents, six PGY-2 residents, and three PGY-3 residents. The training for simulation delivery for EM residents took place at a monthly journal club literature review session, which was held in a classroom on the medical school campus.

**Fig. 1:**
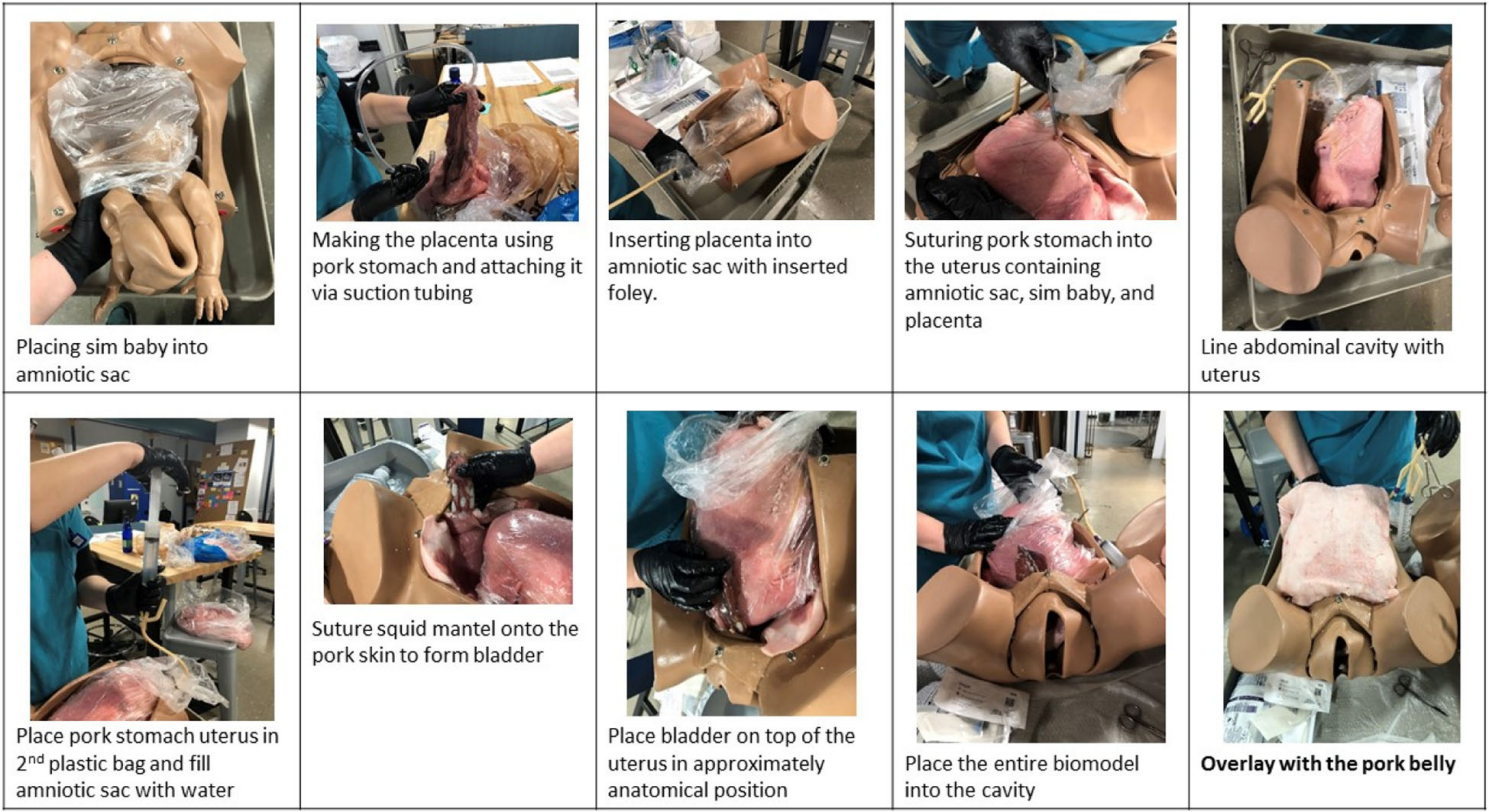
Condensed instruction guide for the hybrid biosimulation model for resuscitative hysterotomy

Two identical task trainers were implemented to allow for simultaneous procedural learning among the learners for the training program. Simulated human placentas, bladders, and uteri were constructed through the use of porcine skin, porcine stomach, and a squid mantle, respectively, which were secured in place with nylon sutures. The amniotic sac was created through the use of a transparent plastic bag that contained a Gaumard S500 Articulating Newborn and was filled with warm water (i.e., the amniotic fluid) through a Foley catheter. Each task trainer model was placed into a Gaumard S500 Childbirth Simulator with an overlying porcine belly to simulate the gravid abdomen. Once the aforementioned models were created, they were mounted on a plastic dolly cart that allowed for ease of transport across pre-clinical environments. Each model required less than 1 h for assembly (Fig. 1) and cost $28.25 to construct (excluding expired hospital supplies and existing Gaumard S500 task trainers, provided in kind by the institutional simulation center) (Table 1). A detailed step-by-step construction guide for the biosimulated resuscitative hysterotomy can be found in Additional file 1: Appendix 2.

**Table 1:** Biosimulation resuscitative hysterotomy model materials and cost

Item	Cost ($)
Pork belly	12 lbs at 3.00/lb (on sale) = 36
Pig skin	3 lbs at 2.50/lb = 7.50
Squid	2 whole squid = 3.00
Pig stomach of adult size, multiple	4 at 2.50/lb = 10
Clear plastic grocery bags	Free from grocery store
Foley catheter × 2	Expired supply (ED)
3-L water	Tap
Gaumard S500 Original Childbirth Simulator (includes non-articulating simulation baby fetus)	Loan from the Rector Clinical Skills and Simulation Center
Gaumard S500 Articulating Newborn	Loan from the Rector Clinical Skills and Simulation Center
Suction tubing	Expired supply (ED)
Umbilical cord tubing from Foley catheter	Expired supply (ED)
Suture (3-0 to 0-0 nylon, any type)	Expired supply (OR)
Laceration tray	Expired supply (ED)
Gloves	Expired supply (OR)
Blunt scissors	Loan from ED
Scalpel 10 blade	Expired supply (ED)
Absorbent floor mats	Expired supply (ED)
PPE (mask, gown, boots)	Expired supply (ED)

*ED* emergency department, *OR* operating room

Emergent hysterotomy was first demonstrated by the content expert (i.e., EM faculty facilitator) and followed by immediate hands-on deliberate practice. Errors in technique were immediately corrected at each step, and the preceding steps were reviewed prior to continuing the procedure. Learners were cycled twice through both simulators with rapid cycle feedback, utilizing the micro-debriefing format (i.e., feedback was delivered immediately as each step in the procedure was performed) [9]. Residents who were not performing the hysterotomy watched their peers, and questions were encouraged. Residents were divided into pairs, and each pair had the opportunity to perform the procedure. The biosimulation models were reset between procedures via reimplantation of the fetus model with placenta inside new plastic bags with all previous lacerations repaired with nylon suture. Formal feedback on the learners’ self-reported confidence and satisfaction levels was solicited at the end of the workshop through the use of survey that was previously used specifically for low-fidelity resuscitative hysterotomy [6]. Quantitative evaluation of the simulated training session was extracted through a 5-item questionnaire using a 5-point Likert-type scale (i.e., from 1, strongly disagree, to 5, strongly agree). Item scores were added for a cumulative total score, with a possible maximum total score of 25 and a minimum total score of 5.

This study was exempt from review by the Thomas Jefferson University Institutional Review Board (IRB), as the resident workshop was part of the EM residency curriculum. Participants provided verbal consent for their anonymous responses to be collected.

## Results

**Fig. 2:**
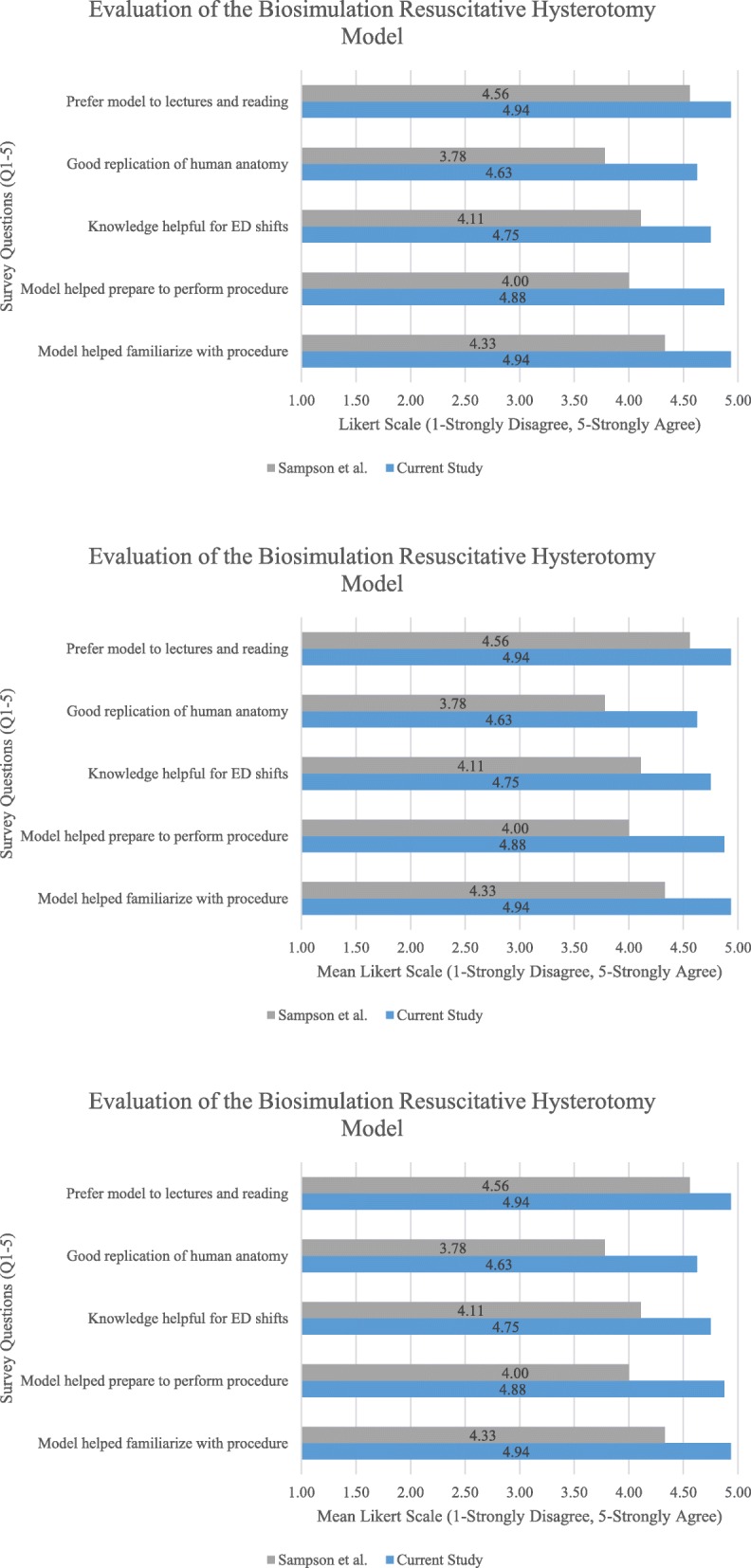
Evaluation of simulation model. All survey questions (1–5) were adapted from Sampson et al.’s [6] survey analysis of a low-fidelity resuscitative hysterotomy model, using a 5-point Likert-type scale

The response rate from resident participants was 100%. Responses were overwhelmingly positive [24.13 (± 1.36)]. Nearly all residents indicated the model was a good representation of human anatomy [4.63 (± 0.62)] and helped them become more familiar [4.94 (± 0.25)] and prepared [4.88 (± 0.34)] to perform resuscitative hysterotomy. Residents reported that knowledge gained would be directly applicable to future clinical shifts [4.75 (± 0.58)]. Participants using the current study model gave overall more positive feedback than the model utilized by Sampson et al.; however, the small sample size (16 in present study and 9 for Sampson et al.) is insufficient to achieve the appropriate level of statistical power for comparison via Student’s *t* tests **(**Fig. 2**)**. Enthusiasm for the course was evaluated using a 3-point scale. Ratings at the time of initial registration indicate medium interest with wide variability [2.38 (0.62)]. At the conclusion of the course, all participants rated their interest in the course as high [3.00 (0.00)]. Ratings for enthusiasm post-course were not significantly higher than pre-course (*t* = 5.37 × 10^−4^, *p* = 0.50). All participants (100%) recommended the training session be available for future emergency medicine residents.

Qualitative feedback from self-written responses highlighted the learners’ appreciation for hands-on practice (81% of resident responses), practice-changing management towards resuscitative hysterotomy (88% of resident responses), and the realistic nature of the tissue-based simulation task trainer (43% of resident responses). Areas for improvement included (1) additional models to allow for repeated practice (i.e., more than two times), (2) ongoing cardiopulmonary resuscitation (CPR) during the procedure to further increase realism, and (3) visual printout of the procedural steps with the model.

## Discussion

Creating an inexpensive, lifelike, simulation training model with tactile realism was a primary goal of this innovative workshop. Previously attempted models have relied on expensive simulation equipment as the basis for their models. One model in the literature [4] is based on the Gaumard NOELLE unit, which costs $4295 at the time of this paper alone (http://www.gaumard.com/s550). By contrast, the simulation pelvis used in this study was much cheaper ($595), including the simulation baby (http://www.gaumard.com/s500), and could be used several times for multiple learners. Though the equipment could theoretically be borrowed from a university simulation center, not all residency programs have access to expensive high-realism simulation models or access to a simulation center for this purpose. Even without the simulated pelvis, this model could easily have been placed in a simple plastic wash basin commonly found in the ED, and the procedure could still have been performed in its entirety. This in turn supports the portability of the model to many different clinical and pre-clinical environments for training.

In addition, the entire cost of the disposable materials in this model was $56.50 for two high-fidelity tissue models. Though this at a glance is more expensive than the materials used in prior attempts ($70 for seven models [6] or $15–20 for one model [5]), the subtle benefit of incorporating animal tissue models is the ability to suture the tissue together post procedure and re-arranging the tissue for the next participant, allowing for the creation of multiple fresh models to allow participants experience the procedure in an RCDP style repetition several times. In a technological age where anatomy labs are becoming more expensive to upkeep and digital applications are taking over standard pedagogy, tactile learning and experience are somewhat lost. As a further attribute, a tissue-based model allows the learner to feel the different layers of anatomy during incision, a sensation that cannot be reproduced with commercial and/or gelatin products.

As a final benefit of the present model, the creation of two models took the study investigators less than 1 h each to construct from start to finish. One model described in the literature [6] requires latex to dry overnight as a single step, in addition to having gelatin set for 2 h at a time. Other published studies [4, 6] do not specify a construction time. The study investigators propose that the quick production and rapid portability across different learning spaces make this an efficacious model for learning resuscitative hysterotomy.

When considering other cesarean simulators currently available on the market or those that allow for such modifications (Table 2), some important key issues arise. Though the other models listed are serviceable for educating general procedure and anatomy, the disadvantage lies in their higher cost and expenditure. In our opinion, only one model is debatably comparable in realism, but is extreme in cost (Operative Experience’s C-Celia - Obstetric Simulator for Fetal Extractions at Cesarean Deliveries, ranging from $13,200 to $19,200). Combining bio tissue or moulage materials with these simulators can result in damage to electronic components, some which may be difficult to sterilize. The current chosen model was easy to outfit with the aforementioned tissue and decontaminate after the session, making it more efficacious in cost, durability, and realism.

**Table 2:** Currently commercially available obstetrical simulation models/task trainers/simulators

Gaumard Advanced Childbirth Simulator CS500 “OB Susie”	$595	https://www.gaumard.com/s500
Simulaids Obstetrical Manikin 180	$660	https://www.simulaids.com/product/110-180
3B Scientific Birthing Simulator Basic	$942	https://www.a3bs.com/3b-birthing-simulator-basic-1020332-p90b-3b-scientific,p_895_28348.html
3B Scientific Birthing Simulator PRO	$1423	h ttps://www.a3bs.com/3b-birthing-simulator-pro-1020333-p90p-3b-scientific,p_895_28349.html
3B Scientific Birthing Simulator	$1945	https://www.a3bs.com/birthing-simulator-1001260-vg395-3b-scientific,p_1453_2262.html
Kyoto Kagaku Vaginal Delivery Assistance Simulator	$4000	https://www.gtsimulators.com/Kyoto-Kagaku-Vaginal-Delivery-Assistance-Simulator-p/kk-mw36.htm
Adam, Rouilly DESPERATE DEBRA	$6400	http://www.adam-rouilly.co.uk/productdetails.aspx?pid=3566&cid
Limbs & Things PROMPT Birthing Simulator - Standard	$5835	https://www.limbsandthings.com/us/our-products/details/prompt-flex-standard
Limbs & Things Caesarean Section Module Prompt Flex(add-on)	+$2370	https://www.limbsandthings.com/us/our-products/details/prompt-flex-cesarean-section-module
Operative Experience C-Celia - Obstetric Simulator for Fetal Extractions at Cesarean Deliveries	$13,300–$19,200	https://operativeexperience.com/fetal-extraction-simulator
Gaumard NOELLE S550	$4295	https://www.gaumard.com/s550
Nasco Life/form Lucy Maternal and Neonatal Birthing Simulator - Basic Lucy	$5900	https://www.enasco.com/p/LF00042U
Laerdal SimMom	$30,000	https://www.laerdal.com/us/products/simulation-training/obstetrics-pediatrics/simmom
Simulaids SMART MOM Basic Birthing Simulator	$30,600	https://www.a3bs.com/smart-mom-basic-birthing-simulator
Gaumard Victoria S2200	$56,500	http://www.gaumard.com/s2200-victoria-childbirth-simulator

The model’s structural advantages were mirrored by the overwhelmingly positive feedback by the EM learner-participants in familiarizing and preparing them for future performance. This study employed the same assessment scale as previously described by Sampson et al.’s [6] low-fidelity resuscitative hysterotomy model, with resounding success. The cumulative satisfaction scores between our model and Sampson’s model were 24.13 and 20.78, respectively. The data from both studies show a preference of hands-on simulation above traditional lectures and reading alone, the improvement in procedural competency, and usefulness for future emergency department shifts. The use of animal tissue also offers both cost-effective and high-fidelity advantage in preparing participants for the techniques necessary in dissecting through actual tissue layers. Furthermore, the RCDP style training was well-received by all participants by allowing them to improve upon their previous mistakes in real time, be offered feedback on their technical skills, and review critical actions before progression. This simulation can be combined with CPR as described by Nadir et al. to further increase the fidelity [5].

With respect to the Accreditation Council for Graduate Medical Education (ACGME) milestones for resident competency, this high-realism task trainer, if fully implemented with the use of high-fidelity simulation techniques (i.e., incorporating confederate actors or environmental props), has the potential to enhance future patient care with respect to the following: Emergency Stabilization (Patient Care 1), Diagnosis (Patient Care 4), General Approach to Procedures (Patient Care 9), Medical Knowledge, and Team Management (Interpersonal and Communication Skills 2) [10]. Residents are challenged to recognize the critical situation of a pregnant mother in extremis; understand the indications to perform the resuscitative hysterotomy; communicate with staff and preceptors about their intent, equipment, and personnel required; and physically perform the procedure in a safe clinical environment. The challenges of this simulation reflect sufficiently the skills needed, stress level, and mental capacity required to manage critical illness under duress.

### Limitations

The study was limited in power due to being at a single center and including a small sample size of EM learners. Despite using an assessment from a previous study, we were unable to determine a statistical significance due to the small sample sizes. Though the results promisingly suggest that a tactile, tissue-based, RCDP simulation can stimulate learner knowledge and enhance practice patterns, more study is needed to adequately demonstrate that this particular format is superior to traditional simulation-based learning and journal club experiences.

## Conclusion

Resuscitative hysterotomy is a high-stakes, low-frequency procedure that demands provider practice and confidence. Review of procedural steps, indications, and materials needed is imperative to the success of this procedure in pregnant patients in extremis. Our hybrid, tissue-based hysterotomy model shows promise in adding the next level of realism for deliberate procedural practice while offering additional utility in cost, reproducibility, and portability to learners of various abilities. Future studies can explore learning benefit differences between using cheaper versus expensive hysterotomy task trainers (i.e., NOELLE) for procedural competency and knowledge retention of emergency hysterotomies. As the medical simulation industry continues to advance, innovative medical moulage is expected to become more readily available and cost-effective, further increasing realism as well as alleviating challenges that may emerge with using bio tissue.

## Additional file

Additional file 1: **Appendix 1.** Detailed step-by-step construction guide to the biosimulation resuscitative hysterotomy model. **Appendix 2.** Post-biosimulation resuscitative hysterotomy survey. (DOCX 1176 kb)

## Abbreviations

ED: Emergency department
EM: Emergency medicine
PGY: Post graduate year
RCDP: Rapid cycle deliberate practice
