# Supplementary material for: A novel biosimulation task trainer for the deliberate practice of resuscitative hysterotomy
Source: Adv Simul (Lond). 2018 Oct 4;3:19. doi: 10.1186/s41077-018-0078-1 (PMC6172835; doi:10.1186/s41077-018-0078-1)
Supplement: Supplementary file 1 — Appendix 1. Detailed step-by-step construction guide to the biosimulation resuscitative hysterotomy model. Appendix 2. Post-biosimulation resuscitative hysterotomy survey. (DOCX 1176 kb) [file 41077_2018_78_MOESM1_ESM.docx]

**Additional file 1: Appendix 1:** Detailed step-by-step construction guide to the biosimulation resuscitative hysterotomy model.

| Insert simulation baby fetus into a plastic bag which will act as the amniotic sac | 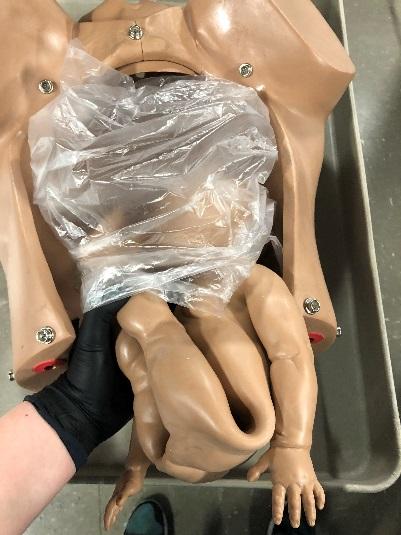 |
| --- | --- |
| Make the placenta: cut suction tubing in two and suture the port end into the pyloric sphincter of the smaller pork stomach.  Option to leave cardiac sphincter and other holes in stomach open (simulating areas of potential torn placenta and prompting learner to sweep uterus for retained pieces of placenta) or suture shut to simulate intact placenta | 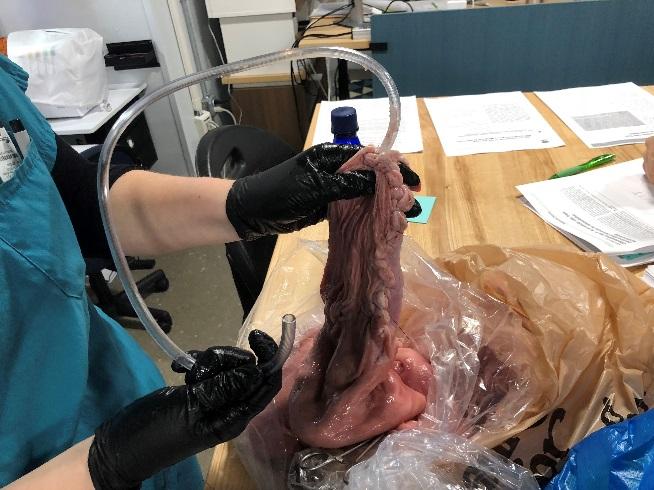 |
| Insert the placenta into amniotic sac in the desired location around simulation baby fetus.  Insert foley 4-5 centimeters into the amniotic sac with bladder opening/balloon end of foley within bag and drainage/balloon ports out of bag | 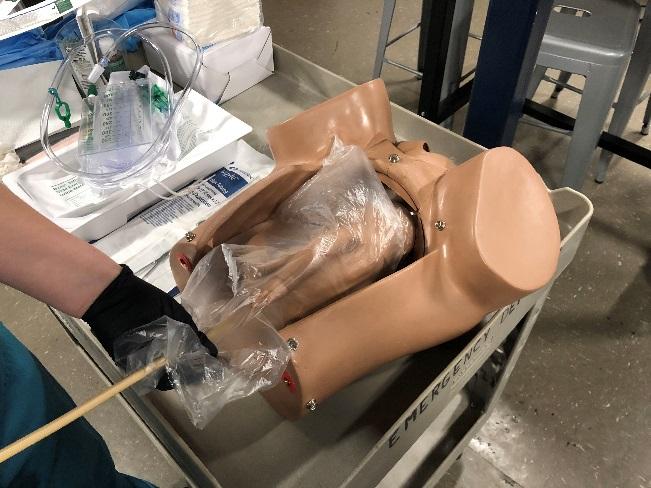 |
| Tie a rubber band by looping many times to keep foley in place | 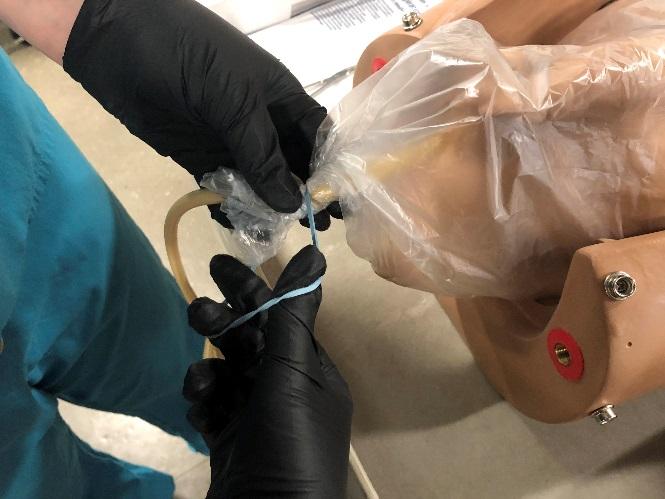 |
| Test for potential fluid leakage by applying traction to foley – if foley slips out easily then continue to loop rubber band to secure it in place.  Inflating the foley balloon will not prevent leakage of your amniotic fluid | 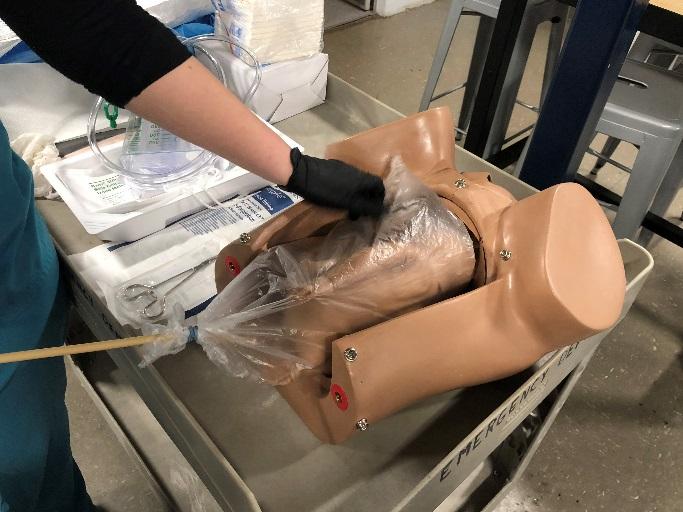 |
| Insert amniotic sac into large pork stomach gently but firmly (best done with an assistant).  You may need to widen the opening of one of the sphincters to get the amniotic sac through, try to minimize the size of said opening | 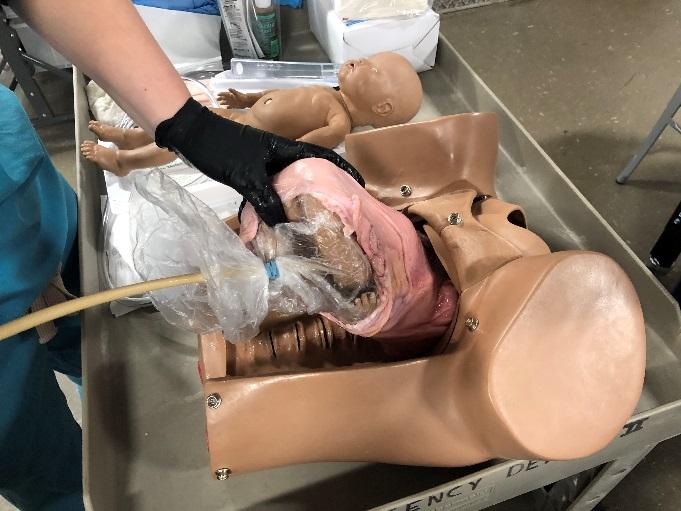 |
| Ensure that the part of the pork stomach intended to become the inferior/pelvic part of the uterus are closed and taught, suture if needed | 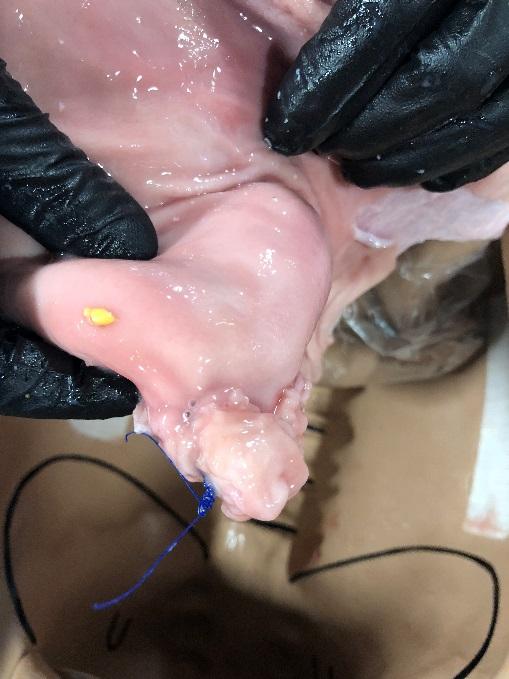 |
| Suture the pork stomach opening so that it is mostly closed and will become taut when water is introduced as amniotic fluid.  It is not necessary to completely close the stomach as this part is intended to become the superior uterus thus will be hidden under “abdominal wall”.  Learners are not intended to manipulate this part of the uterus | 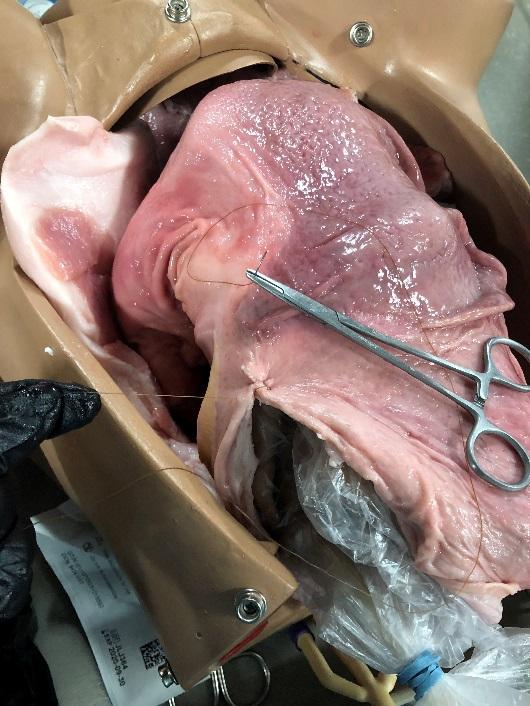 |
| Suture pork stomach into a uterus containing amniotic sac and simulation baby with foley for introduction of amniotic fluid | 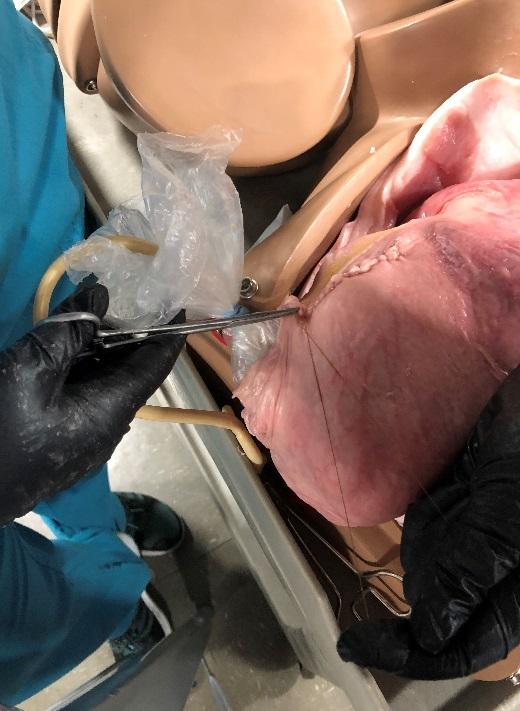 |
| Line abdominal cavity with pork skin (arrow).  Check the sizing of the uterus to the maternal abdomen/pelvis.  Ideally the fundus will high so that it will be palpable above the umbilicus when the pork belly layer is laid down as maternal abdominal wall.  Add packing with additional meat to the pelvis as needed to achieve a high fundus | 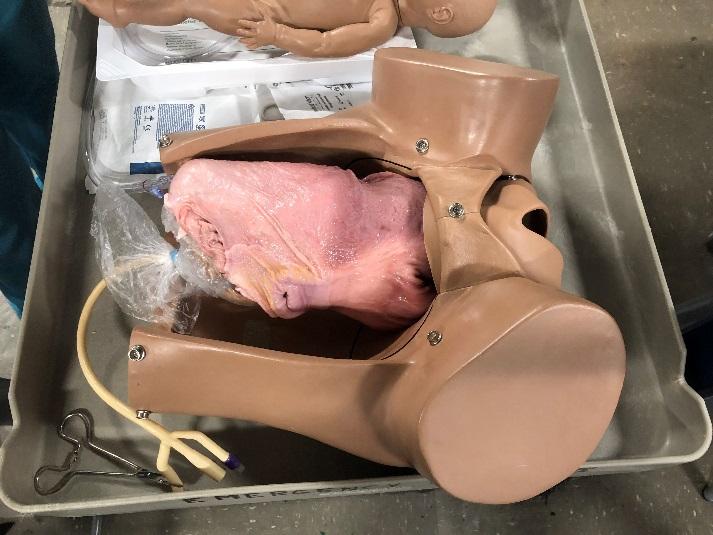  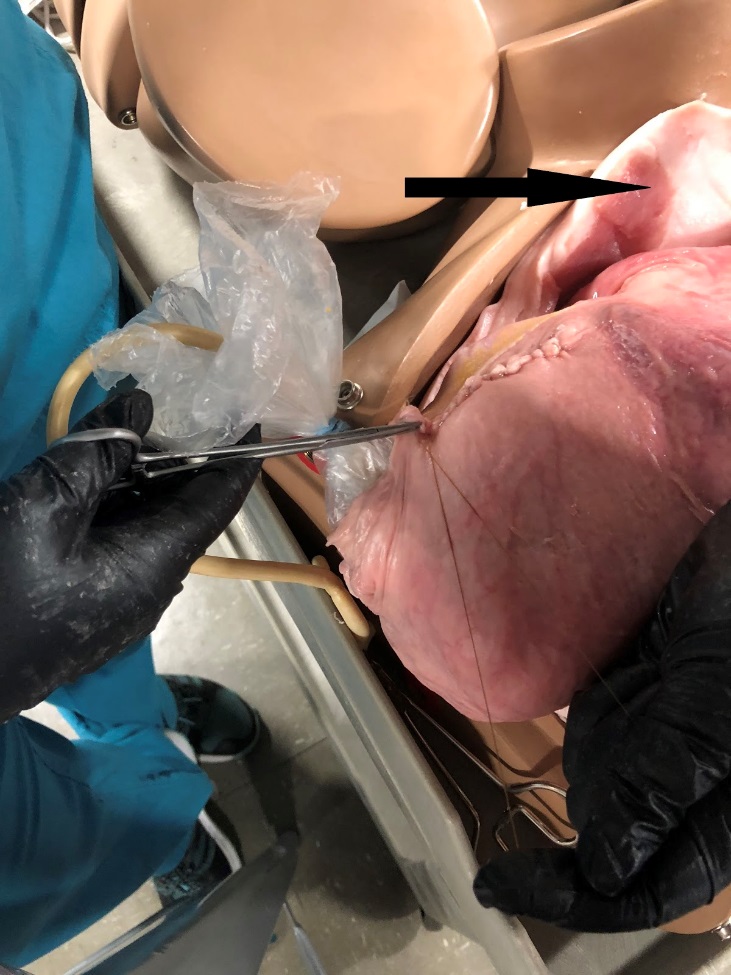 |
| Put pork stomach uterus into second plastic bag which will act as peritoneum and place into abdominal cavity | 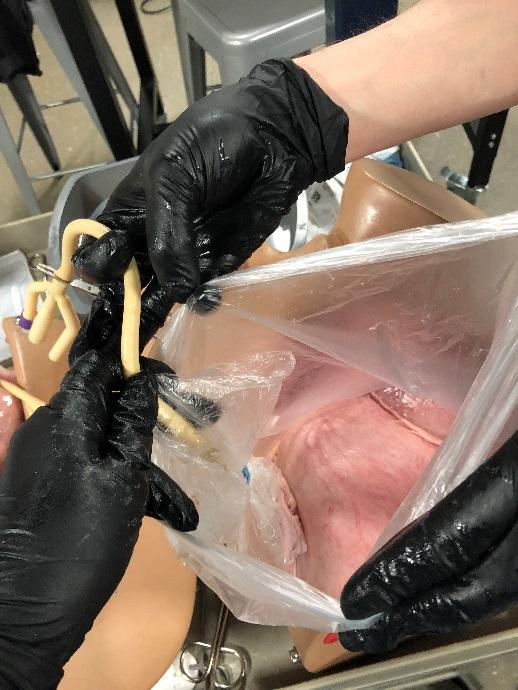 |
| Fill amniotic sac with water using a Toomey syringe attached to bladder port of foley catheter | 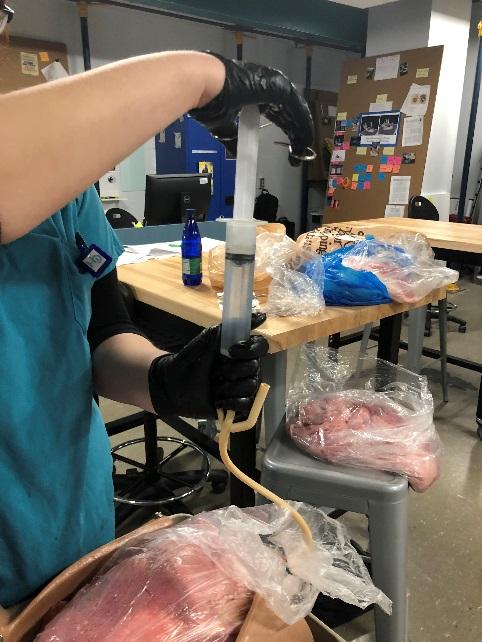 |
| To maintain the fluid inside the amniotic sac the foley can be clamped or a bag can be attached and clamped off.  If the bag is used then additional fluid can be loaded into bag for additional volume during the first learner’s procedure or can be at-the-ready for a second learner’s procedure after repair of the model | 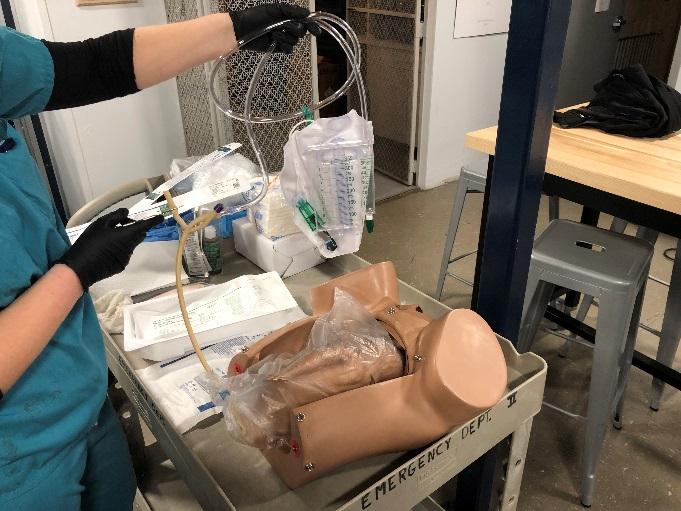 |
| Suture the squid mantel onto the pelvic part of the pork skin lining of the abdominal cavity to form the bladder | 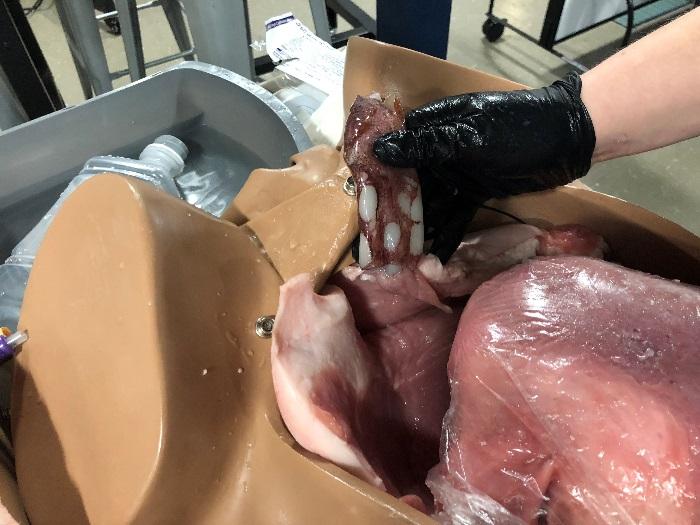 |
| Place bladder on top of the peritoneal layer and uterus in approximately anatomical position so that learners will encounter it during procedure.  Bladder can be sutured shut and filled with fluid if desired | 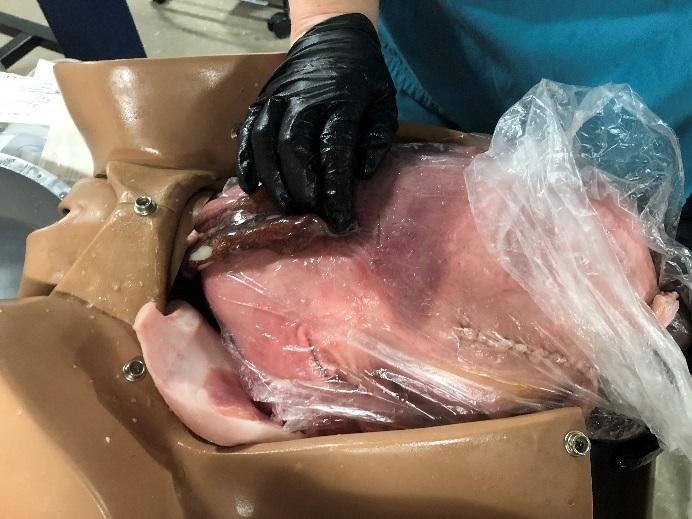 |
| 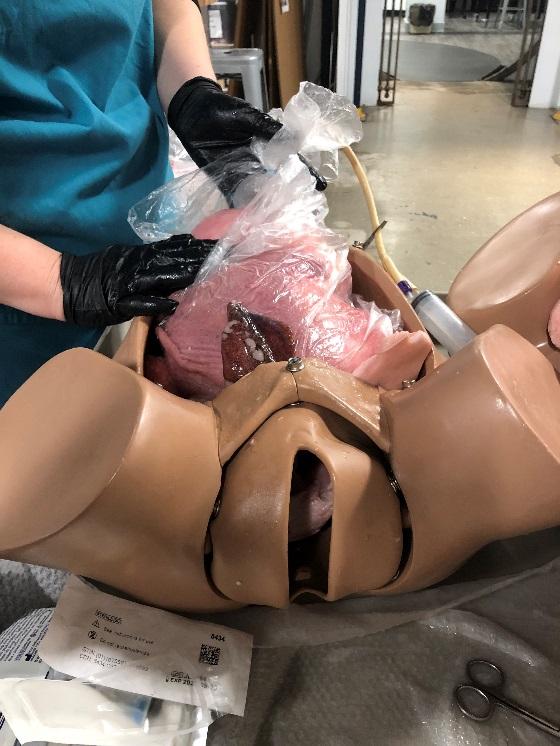 | 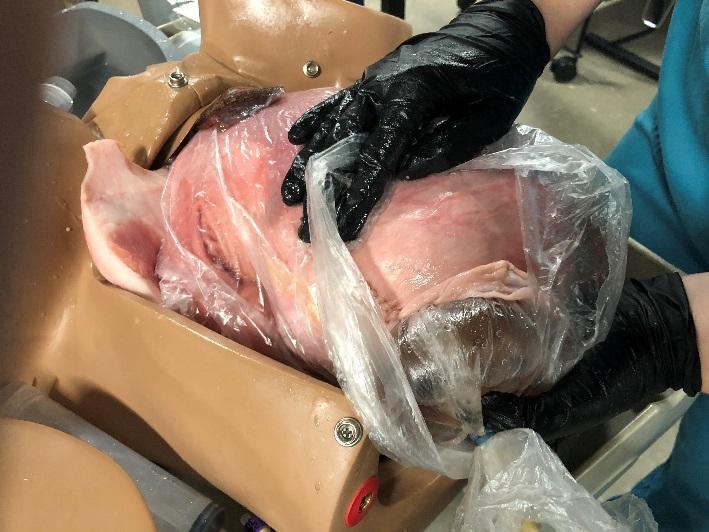 |
| Lay pork belly over completed abdominal cavity to create the maternal abdominal wall.  The superior edge of the uterus can be bolstered by the weight of additional pork belly tissue extending over top of the model to help shift amniotic fluid inferiorly.  Tuck foley catheter under edge of additional tissue | 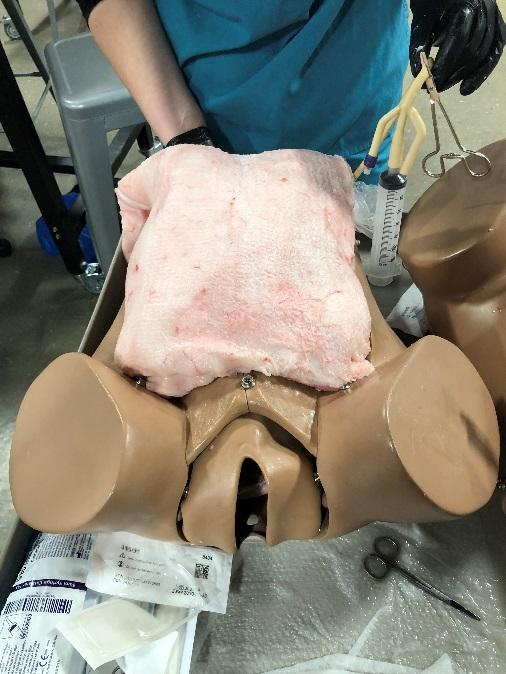 |

**Additional file 1: Appendix 2:** Post-biosimulation resuscitative hysterotomy survey

*Please complete the evaluation form as indicated. If the statement is not applicable, please mark the ‘N/A’ column. Please place all of the forms facedown at the front of the desk after completion. Thank you very much.*

Please fill out the following questions to describe **yourself**:

1. What is the current level of your training *(please mark with ‘X’)*:
   1. ___ PGY 1 ___ PGY 2___ PGY 3___ PGY 4
2. Have you ever **participated** in a journal club before: ⃝ Yes ⃝ No
3. Have you ever **participated** in a procedural simulation teaching session before: ⃝ Yes ⃝ No
4. Have you participated in a journal club with procedural teaching?: ⃝ Yes ⃝ No
5. Have you ever performed a perimortem cesarean section before?: ⃝ Yes ⃝ No

**Instruction**: Please indicate your level of agreement regarding the simulation

|  | Strongly Disagree | Disagree | Neutral | Agree | Strongly Agree | N/A |
| --- | --- | --- | --- | --- | --- | --- |
| The model helped familiarize you with peri-mortem cesarean section | ⃝ | ⃝ | ⃝ | ⃝ | ⃝ | ⃝ |
| This model helped you better prepare to perform this procedure in the future | ⃝ | ⃝ | ⃝ | ⃝ | ⃝ | ⃝ |
| The knowledge from this session would help during your emergency department shift | ⃝ | ⃝ | ⃝ | ⃝ | ⃝ | ⃝ |
| The model was a good replication of human anatomy | ⃝ | ⃝ | ⃝ | ⃝ | ⃝ | ⃝ |
| This session enhanced learning more than traditional lectures and reading alone | ⃝ | ⃝ | ⃝ | ⃝ | ⃝ | ⃝ |

End of the Course Evaluation:

1. Your level of enthusiasm for taking this course at the time of initial registration

⃝ Low ⃝ Medium ⃝ High

1. Your level of enthusiasm for the course at the conclusion of the course

⃝ Low ⃝ Medium ⃝ High

1. Considering your experience with this course, would you recommend it to other students?

⃝ Yes ⃝ No

1. What did you like most about this training?
2. What aspects of the training could be improved?
3. How do you hope to change your practice as a result of this training?
4. What additional challenges would you like to see in the future?
5. Please share other comments or expand on previous responses here:
